# Experiences and perspectives during the transition from paediatric to adult care in type 1 diabetes mellitus: systematic review of qualitative studies

**DOI:** 10.1007/s00431-025-06303-5

**Published:** 2025-07-15

**Authors:** Ana Toledo-Chavarri, Alezandra Torres Castaño, María Padilla Ruiz, Cristobalina Rodríguez-Álvarez, Angeles Arias, Miguel García-Hernández

**Affiliations:** 1 Instituto de Estudios Sociales Avanzados (IESA), Consejo Superior de Investigaciones Científicas (CSIC), Network for Research On Chronicity, Primary Care, and Health Promotion (RICAPPS), Córdoba, Spain; 2 Network for Research On Chronicity, Primary Care, and Health Promotion (RICAPPS), Canary Islands Health Research Institute Foundation, Carretera Gral. La Cuesta, 75-77, 38320 La Laguna, Santa Cruz de Tenerife, FIISC Spain; 3 Network for Research On Chronicity, Hospital Universitario Costa del Sol, Primary Care, and Health Promotion (RICAPPS), Marbella, Spain; 4 Department of Preventive Medicine and Public Health, University of La Laguna, 38200 Canary Islands, Spain; 5 Gerencia de Atención Primaria del Área de Salud de Tenerife, Network for Research On Chronicity, Primary Care, and Health Promotion (RICAPPS), Unidad Docente de Atención Familiar y Comunitaria La Laguna-Tenerife Norte, Santa Cruz de Tenerife, Spain

**Keywords:** Diabetes mellitus type 1, Transition to adult care, Qualitative evidence synthesis, Adolescent

## Abstract

When Type 1 diabetes mellitus (T1DM) patients transition from paediatric to adult healthcare providers, the risk of deteriorating health outcomes rises. The aim of this study is to integrate the experiences and perspectives regarding interventions for the management of T1DM during the transition from paediatric to adult care, from a patient and professional perspective. A systematic review was conducted with a framework analysis guided by the Cochrane Qualitative and Implementation Methods Group approach. Three online databases (Medline, Embase and Web of Science) were searched. We included qualitative articles that were available in English or Spanish. Twenty-five references met the inclusion criteria of the study and were included. Four main themes were identified: (a) teen experience, (b) acceptability, (c) implementation considerations and (d) equity.

*Conclusion*: During the care transition, adolescents with T1DM undergo a critical phase of increasing care independence in which fostering autonomy and strengthening continuity of care may achieve a more satisfying care.

**Table:** 

**What is Known:** *• Transitions from pediatric to adult healthcare providers are associated with an increased risk of adverse health outcomes.*	
**What is New:** *• This study highlights that enhancing the transition process requires improved information transfer, reinforced continuity of care, and effective coordination among healthcare providers.*

**Supplementary Information:**

The online version contains supplementary material available at 10.1007/s00431-025-06303-5.

## Introduction

T1DM is a chronic condition characterised by an immune-mediated destruction of pancreatic beta cells and absolute insulin deficiency, whose pathogenesis is principally provoked by the interaction of known genetic, but also environmental risk factors [1]. Several non-specific environmental factors such as being overweight, contracting infections or experiencing stress that occur in genetically predisposed individuals alone or in combination, end up generating the cascade of reactions that will determine the secondary destruction of beta cells [2].

Typically, the incidence of T1DM peaks around puberty, with the presence of autoantibodies against pancreatic islet antigens some years before the onset of the disease [3]. The prevalence is increasing worldwide and a higher incidence has also been found among youth in the USA in the last decade [4]. In the Spanish working population, the rate is 0.3% [5], and, in some regions of Spain, among young individuals (under 15 years of age), it is estimated to be around 0.1% [6]. Even though there is no shortage of hypotheses to explain, there is a great heterogeneity of incidence rates between countries, with the highest rates found in Scandinavian and European countries. By contrast, in Asian countries, T1DM is considered a rare disease [7].

The presence of T1DM represents, not only a substantial clinical and public health burden, but also a high impact on the quality of life of both patients (adults and paediatric age) and their caregivers, which is closely related to the severity of the disease, determined by poor glycemic control and the presence of complications and comorbidities related to diabetes [8].

From a social and health system point of view, T1DM is a disease with a high economic impact due to both acute complications (such as hypoglycaemia or ketoacidosis) and chronic complications, raising public health spending and employment resources in these patients, as well as the care burden of informal caregivers [9]. The total cost of the disease, taking into account the direct and indirect costs, comes to around 27,247 € per patient of paediatric age per year [9].

In order to improve the health status of patients with T1DM and reduce the number of associated complications, it is not enough to intensify pharmacological treatment. Positive health behaviours and psychological well-being have been identified as crucial for achieving diabetes treatment goals [10]. Following the recommendations of the American Diabetes Association, “all people with diabetes should participate in diabetes self-management education and support to facilitate the knowledge, decision-making, and skills mastery for diabetes care” [10]. However, there are some crucial moments to evaluate the need for education and support, among which transitions in life and care stand out [10].

In fact, as late adolescents and young adults with T1DM reach adulthood, the risk of worsening health outcomes increases [11]. Data obtained from studies carried out in most western countries reported that the majority of patients with T1DM do not reach the proper metabolic control target [12]. Particularly in paediatric and young age, there is great difficulty in adhering to healthy eating patterns [13] which may contribute to inadequate metabolic control. Likewise, when patients transition from paediatric to adult healthcare providers, there is an increased risk of DM-related hospitalisations, especially when interruption of care occurs [14].

This systematic review aimed to integrate the experiences and perspectives related to acceptability, implementation and equity of the transition from paediatric to adult care in T1DM from patients, their parents and professional perspectives.

## Material and methods

### Design

A systematic review was conducted with a framework analysis guided by the Cochrane Qualitative and Implementation Methods Group approach [15], and the reporting of the results has followed the Preferred Reporting Items for Systematic Reviews and Meta-Analyses (PRISMA) statement [16].

The “Enhancing Transparency in Reporting the Synthesis of the Qualitative Research” (ENTREQ) checklist was used to guide the reporting of this Qualitative Evidence Synthesis (QES) [17]. The framework analysis used the categories of acceptability, implementation, and equity from the *Evidence To Decision framework* (EtD) from *Grading of Recommendations Assessment, Development and Evaluation* (GRADE) [18], [19]. This framework establishes research questions that can guide the comprehension of each category (Table 1).

**Table 1:** Frameworks research questions

Research questions related to acceptability, implementation and equity	
Experiences What are the experiences related to the intervention?	• Are there stakeholders experiences in relation to the intervention?
Acceptability Is the intervention acceptable to key actors?	• Are there key stakeholders that would not accept the distribution of the benefits, harms and costs?
	• Are there key stakeholders that would not accept the costs or undesirable effects in the short term for desirable effects (benefits) in the future?
	• Are there key stakeholders that would not agree with the values attached to the desirable or undesirable effects (because of how they might be affected personally or because of their perceptions of the relative importance of the effects for others)?
	• Would the intervention adversely affect people’s autonomy?
	• Are there key stakeholders that would disapprove of the intervention morally, for reasons other than its effects on people’s autonomy (e.g. in relation to ethical principles such as no maleficence, beneficence or justice)?
Implementation considerations Is the intervention feasible to implement?	• Is the intervention or option sustainable?
	• Are there important barriers that are likely to limit the feasibility of implementing the intervention (option) or require consideration when implementing it?
Equity What would be the impact on equity?	• Are there plausible reasons for anticipating differences in the relative effectiveness of the option for disadvantaged groups or settings?
	• Are there different baseline conditions across groups or settings that affect the absolute effectiveness of the intervention or the importance of the problem for disadvantaged groups or settings?
	• Are there important considerations that should be made when implementing the intervention in order to ensure that inequities are reduced, if possible, and that they are not increased?

Adapted from Moberg 2018(Moberg et al., 2018)

### Search strategy

We searched MEDLINE, EMBASE and Web of Science from 1900 to April 2021. The search strategy was developed initially in MEDLINE using controlled vocabulary and free text terms and then adapted for each of the other databases. The search was restricted to studies published in English or Spanish. Full search strategies for all database searches are available in supplementary material (see Supplementary material 1).

Two reviewers screened independently and in duplicate the titles and abstracts of all the retrieved citations. Then the full texts of all citations that appeared to fulfill the pre-determined selection criteria were read and evaluated for inclusion, again, independently and in duplicate by the reviewers. When doubts or disagreements occurred, they were resolved through discussion within the research team until consensus was reached.

### Eligibility criteria

References were included if they addressed the objectives of this review, used qualitative techniques and reported qualitative findings separately. Studies in languages other than Spanish and English were excluded. Disagreements were solved by discussion within the team. The complete inclusion and exclusion criteria can be found in Table 2.

**Table 2:** Inclusion and exclusion criteria for the selection of the studies

	**Population**	**Context**	**Findings**	**Design**
Inclusion	People with a diagnosis of T1DM	Transition period from paediatric to adult care in T1DM	Experiences or trajectory of care, acceptability of the interventions defined in each question, context of implementation, acceptability and equity	Qualitative studies or mixed methods studies reporting results separately
Exclusion	Other populations (diabetes type 2, gestational diabetes, healthy population, etc.)	Any other moment of T1DM care	Any other finding	Randomised clinical trials, non-randomised clinical trials, quasi-experimental studies, narrative reviews, editorials, letters to the editor and abstracts

### Data abstraction

A thematic synthesis adapted from Thomas and Harden [20] was carried out within the framework analysis using the software Nvivo12® to support the process. The whole team independently coded a sample of two studies each to develop an initial codebook based on the above-mentioned framework for this study, extended with deductive codes from the included studies. The codebook was then discussed within the team and used to code the rest of the studies. The final version of the codebook can be seen in Table 3. Two independent reviewers extracted all relevant qualitative findings in each of the studies, and descriptive themes were generated and discussed among the whole team. A finding reporting form was prepared according to the selected framework and the cited categories of disease management, acceptability, implementation and equity.

**Table 3:** Code book

Category	Codes	Description
Theme: Experiences		
Disease management	Privacy	Experiences related to the privacy of their illness and its stigmatisation
	Fear of hypoglycaemia	Treatment or disease management experiences related to fear of hypoglycaemia
	Device use	Experiences with glycemic control and treatment devices
	Disease burden	Experience and thoughts about feeling chronically ill
	Diagnosis of the disease	Experience at the time of diagnosis of the disease
	Complications related to the disease	Occurrence of complications related to disease progression
Daily life	Daily life activities	Experiences and barriers to carrying out daily activities in relation to their illness
	Maternity	Perceptions and expectations in relation to the beginning of adult life and the possibility of motherhood
	Work life	Perceptions and expectations in relation to the beginning of adult life and work activity
	Diabetes camps	Perceptions regarding visiting camps for young diabetics
Theme: Acceptability		
Values and preferences	Patient perception	Adolescent perceptions of the transition of care
	Parents perception	Parents perceptions of the transition of care
	Health workers perception	Health workers perceptions of the transition of care
	Support between patients	Contact, communication and support between patients in the transition
Interaction with health workers in the transition	Proximity to healthcare	Patients’ experiences of individual healthcare and desire for support
	Satisfaction of care	Patient experiences in relation to the care received and perception of its quality
Patient autonomy	Information needs	Experience and need for information in the transition of care, including both patients and parents
	Treatment negotiation	Risk–benefit balance of health interventions in relation to patient preferences
	Identity	Patient's needs to feel recognised and independent
	Parental involvement	Frequency of attendance at consultation, role and decision making
Organisation of services	Organisational change needs	Thoughts on generating changes in the organisation of care during the transition
	Knowledge of health workers about the organisation of services	Knowledge of caregivers about the care that patients will receive
	Bridge activities	Perception of patients and healthcare providers on bridging activities
	Suggestions for improvement	Suggestions and opinions from patients and healthcare providers to improve care
Temporary adaptation	Completion of paediatric care	Experiences and recommendations for the continuity of doctor-patient communication when leaving paediatric care
	Time of transition	Perceptions and experiences of caregivers and patients about the age when the transition takes place
Theme: Implementation considerations		
Continuity of care	Visibility	Whether or not the patients are known by the center where they will begin receiving care
	Care references	Continuity of care with the same paediatric professionals in the transition to adulthood
	Care overlap	Experience in relation to the continuity of care by the same professionals in the transition to adult care
	Attendance compliance	Strategies implemented when an appointment is missed and opinions on improving adherence to consultations
Feedback	Communication between healthcare workers	Communication between healthcare professionals, especially between those responsible for paediatric and adult care
	Feedback	Opinion of healthcare professionals on the creation of feedback strategies after the transition of care
Theme: Equity		
Access	Barriers to access	Difficulties and awareness that patients encounter to access to care
	Barriers to communication	Difficulties that patients encounter with communication and understanding of the doctor or nurse consultation
	Support Resources	Interaction with support services and communication between health professionals and adolescents

### Synthesis and quality appraisal

The characteristics of individual studies were collected in a table specifically designed for this review. The table includes first author, year of publication, country, aim of the study, qualitative study design, sample, setting and methodological limitations of the study. The CASP checklist was used as a critical appraisal tool to assess the methodological quality of the studies [21].

## Results

**Fig. 1:**
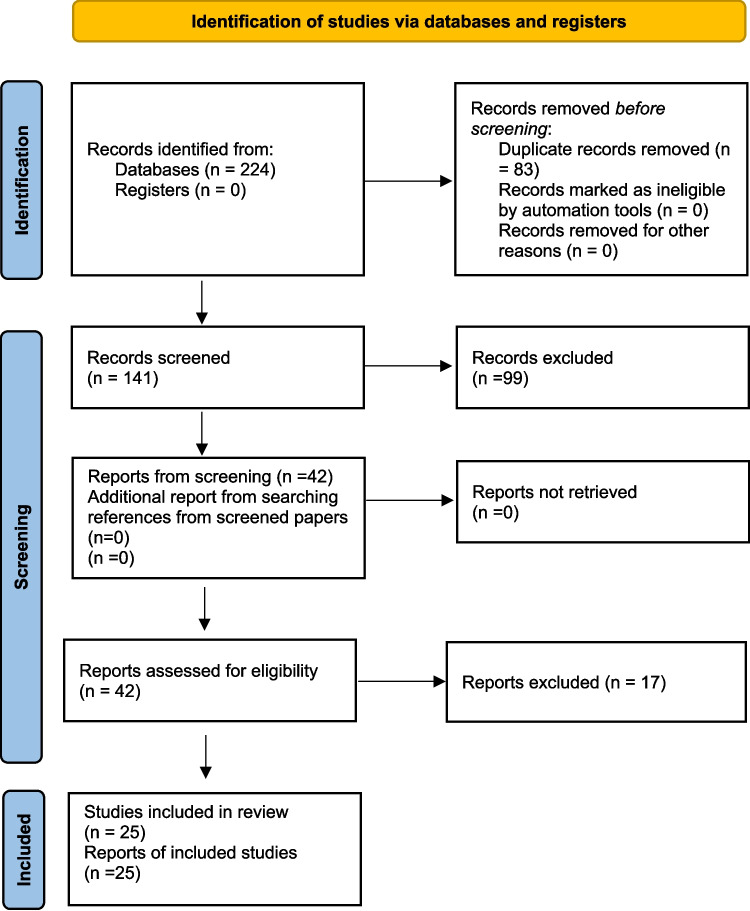
Results of the literature search and study selection process

The results of the literature search and study selection process are shown in Fig. 1 [22]. Identified articles, once the duplicates were eliminated, were one hundred and forty-one. After the first screening, the articles reviewed in full text were forty-two, of which twenty-five were finally eligible for inclusion according to pre-established selection criteria.

The main characteristics of the participants, studies and interventions are summarised in Table 4. Studies were variable in terms of setting, aim, qualitative design and population. The vast majority of them were set in western high-income countries. Nine studies took place in the USA, five in Canada, four in Australia, three in the UK, two in Sweden, one in Taiwan, one in Norway and one in Ireland. The population included a wide range of T1DM patients from a very young age to those who have already completed the transition and were seen in adult consultations. Patients’ caregivers and health workers were also included. The quality assessments of the studies are also summarised in Table 4.

**Table 4:** Characteristics of the studies

**Author, year and country**	**Aim of the study**	**Qualitative study design**	**Sample (*n*)**	**Setting**	**Methodological limitations (CASP)**
D. Allen, Channon, Lowes, Atwell, and Lane, 2011, UK	To explore the experiences of young people and their carers during the transition from childhood to adult diabetes services	Semi-structured interview	138 T1DM patients and 118 caregivers	Five different diabetes services in the UK	Reflexivity has not been reflected upon upon nor is it fulfilled; Data analysis is not described in depth; The presentation of the results is not entirely clear
Davina Allen et al., 2012, UK	To identify the continuity mechanisms essential for a smooth transition from childhood to adult diabetes care, the service components through which they can be achieved, and their interrelationships in different contexts	Realistic evaluation	38 health providers, 46 T1DM patients and 39 “caregivers”	Diabetes services in the UK	Reflexivity has not been reflected upon upon nor is it fulfilled
Butalia, McGuire, Dyjur, Mercer, and Pacaud, 2020, Canada	To learn how to improve the transition of youth with type 1 diabetes from paediatric to adult diabetes care from the perspective of patients and parents	Focus group	11 T1DM patients and 3 parents	Alberta Children’s Hospital. Calgary, Alberta	The participant selection strategy and the context of selection is not clearly defined; Reflexivity has not been reflected upon nor is it fulfilled
Chiang et al., 2020, Taiwan	To explore the life experiences of patients with type 1 diabetes in the transition from adolescence to adulthood	Semi-structured interview	14 young adults (between 16 and 25 years old) with T1DM	Division of Metabolism and Endocrinology of the Department of Paediatrics of one of the leading referral hospitals in Taiwan	The flexibility between researcher and study object is not analysed
Garvey et al., 2014, USA	To explore the experience of transition from paediatric to adult diabetes care reported by emerging adults with type 1 diabetes (T1D) after transition, with a focus on preparing for actual transfer of care	Thematic analysis	26 T1DM patients	Adult Diabetes Clinic at a Comprehensive Diabetes Center in Boston, MA, USA	No reflection on the relationship between the researcher and the object of research (reflexibility) is not fulfilled
Harris et al., 2020, Australia	To explore health professionals’ experiences and perceptions of the support available to young adults with T1D during the transition period	Semi-structured interview	16 health professionals	Public health service provider in Australia	Snowball sampling may have generated sampling bias, and the recruitment methods employed depended on self-selection. The findings were also derived from health professionals employed by a single public health care provider. No reflexibility analysis is performed
Hilliard et al., 2014, USA	To understand the concerns, expectations, preferences, and experiences of teens and parents before transition and young adults after transition	Mixed methods	Non-reported	Paediatric tertiary medical center in the MidAtlantic region of the United States	Reflexivity has not been achieved and is not fulfilled. They have not taken into account ethical aspects
Holtz et al., 2020, USA	To describe and explore adolescents’ and their parents’ perspectives on transition through the transactional model of stress and coping	Semi-structured interview	12 T1DM patients and 9 parents	Local Juvenile Diabetes Research Foundation. Michigan State University. USA	The participant selection strategy and the context of the selection is not clearly defined. Reflexivity has not been achieved and is not fulfilled. The sample size was small and participants were self-selected. Most of the participating fathers were mothers and only those who stated that they were involved in care were interviewed. It does not discuss reflexibility.)
Iversen, Kolltveit, Hernar, Mårtensson, and Haugstvedt, 2019, Norway	To e xplore how young adults with type 1 diabetes experienced the transition from paediatric to adult health care services	Semi-structured interview	11 T1DM patients	Outpatient clinic in western Norway	Some participants declined to participate, which led to a reduced sample size. Reflexibility is not discussed
Leung, Tang, Lim, Laffel, and Amed, 2021, Canada	To explore patient expectations in a program designed for transition to adult care	Focus group	22 T1DM patients	Urban Academic Pediatric Hospital within a universal health system that does not currently have a transition program for adolescents. British Columbia, Vancouver, Canada	Reflexivity has not been achieved. (Participation rate was low (23%), and sex and age characteristics between participants and non-participants were not significantly different.)
Lundin, Danielson, and Öhrn, 2007&Lundin, Öhrn, and Danielson, 2008, Sweden	To explore how caregivers manage the transition process from paediatric to adult diabetes outpatient clinic and describe their perception of adolescent needs during this process, describe the culture of care in paediatric and adult diabetes outpatient clinics and the implications for adolescent care in those settings	Participant observation and semi-structured interview	61 [51 patient observations and 10 professional interviews (7 nursing and 3 medicine	Four different outpatient clinics in Sweden, two in paediatrics and two in adult diabetes care	Reflexivity has not been achieved and is not fulfilled
Nakhla, Bell, Wafa, and Dasgupta, 2017, Canada	To determine the adequacy of paediatric transitional care structures and to explore the perceptions of paediatric transitional care professionals about this transition period	In-depth interviews	8 paediatric care professionals	Pediatric Diabetes Care Centers in Quebec, Canada	No reflexibility analysis is performed
Ng, Crowe, Ball, and Rasmussen, 2017, Australia	To identify the health and wellness needs of young adults in order to develop appropriate solutions for diabetes self-management	Semi-structured interview	13 T1DM patients	Web-site with resources for diabetes. Australia	No reflexibility analysis is performed
Perry, Lowe, Steinbeck, and Dunbabin, 2012, Australia	To describe the healthcare experiences of young adults with type 1 diabetes accessing diabetes services in rural areas of New South Wales, Australia	Semi-structured interview	26 T1DM patients	Diabetes services in rural areas of New South Wales, Australia. Year 2008	Reflexivity has not been reflected upon and is not fulfilled
Pierce et al., 2017, USA	To identify the content domain for developing a multidimensional measure of health care transition outcomes	Interview	10 patients with T1DM, 9 parents, 9 paediatric healthcare workers, 8 adult healthcare providers and 9 clinicians and researchers with experience in the transition of care (expe	Pediatric health system serving the Delaware Valley, North Florida, and Central Florida	The participant selection strategy and the context of the selection is not clearly defined. Reflexivity has not been achieved and is not fulfilled. They have not taken into account ethical aspects
Price et al., 2011, UK	To evaluate the diabetes model of the “transition path” through interviews with young people who have experienced it firsthand	Thematic framework analysis of interviews	11 T1DM patients	NR	The participant selection strategy and the context of the selection is not clearly defined. Reflexivity has not been achieved and is not fulfilled. They have not taken into account ethical aspects
Pritlove, Markowitz, Mukerji, Advani, and Parsons, 2020, Canada	To know the perspectives of parents of young adults with T1D on the management of the disease	Semi-structured interview	16 parents of young adults with T1DM	Two urban diabetes clinics for young adults and through a national diabetes charity	Interviews were conducted mostly with mothers. Reflexibility is not discussed
Pyatak et al., 2014, USA	To examine the challenges that contribute to interruptions in care during the transition from paediatric to adult care among young adults with type 1 diabetes who are primarily from ethnic minority groups and have low socioeconomic status	Semi-structured interview	20 T1DM patients	Southern California, USA. Young adults with type 1 diabetes with a history of loss of follow-up, ethnic minorities, and low resources	The participant selection strategy and the context of the selection is not clearly defined. Reflexivity has not been achieved and is not fulfilled. They have not taken into account ethical aspects
Ramchandani, Way, Melkus, and Sullivan-Bolyai, 2019, USA	To explore developmental, situational, and organisational challenges experienced during transition by a diverse group of emerging adults	Focus groups and individual interview	21 T1DM patients	Paediatric and adult diabetes clinics of an urban academic medical center	There were complications in the recruitment of young adults. No reflexibility analysis is performed
Rasmussen, Ward, Jenkins, King, and Dunning, 2011, Australia	To identify life transitions that may affect diabetes self-management among young adults with type 1 diabetes and their coping strategies during transition events	Semi-structured interview	20 T1DM patients	Adults aged 18–38 years, with DMT1 and living in Victoria, Australia	Reflexivity has not been achieved and is not fulfilled
Tomette, Henderson, Hass, Carson, & King, 2020, USA	To examine parental and caregiver distress among families caring for children with type 1 diabetes as the child transitions into adulthood	Mixed inductive-deductive qualitative research design	11 caregivers (relatives)	Choctaw Nation of Oklahoma, USA	The participant selection strategy and the context of the selection is not clearly defined; Reflexivity has not been achieved and is not fulfilled. They have not taken into account ethical aspects. Data analysis was not rigorous
Tremblay, Ruiz, Buccigrosso, Dean, and Garvey, 2020, USA	To explore expectations for transition to adult care and experiences with transition planning among adolescents and young adults with type 1 diabetes and an A1C of 0.9% in a U.S. paediatric tertiary care facility	Semi-structured interview	13 T1DM patients	A comprehensive paediatric diabetes center operating within a paediatric referral hospital in Boston, MA, without a formal transition program	Reflexivity has not been achieved and is not fulfilled
Walsh, Wynne, O’Donnell, O’Hara, and Geoghegan, 2018, Ireland	To describe the perceptions of young adults, their parents, and health professionals, of the transition process for young adults with type 1 diabetes	Semi-structured interview	6 T1DM patients,, 7 parents DMT1 patients, 7 health care professionals	Paediatric clinic. Ireland	The data are not considered generalisable beyond the west of Ireland. An in-depth analysis of the results is not presented. There is no reflexibility analysis
Williams et al., 2021, Canada	To determine how the transition is occurring in the jurisdiction and identify methods to improve clinical services for paediatric patients with a chronic illness during their transition to adult care	Mixed methods approach	NR	Newfoundland and Labrador, Canadá	The participant selection strategy and the context of the selection is not clearly defined. The data collection techniques used are not entirely consistent with the research question and the method used and data collection is not justified. Reflexivity has not been achieved. They have not taken into account ethical aspects. Data analysis was not rigorous

–

The most relevant themes that emerged in the thematic analysis of the data were:

### Adolescents and young adults with diabetes type 1 experiences at the time of the transition to adult care

Diabetes type I1in adolescence is accompanied by stigma, negative social stereotypes, exclusion, blame and discrimination, and leads to avoidance in disclosing their disease to other people [23, 24]. They express anger at having to deal with this condition for the rest of their lives [25], often requiring a complete restructuring of family life [26].

Nevertheless, during adolescence, diabetic patients tend to increase their involvement in self-care. Patients who transition from adolescence to early adulthood become increasingly aware of their own responsibilities and leave behind their parent’s care. It is known as a conflicting period when patient preferences could collide with the physician’s recommendations [24]. Sometimes patients can change their care routines based on their feelings, increasing the risk of adverse events [24]. Blood glucose control can be challenging [24] as competing life priorities emerge [27]. In addition, patients must juggle multiple commitments and competing life priorities, making it difficult to balance proper healthcare [28] [29]. Alongside general worries such as work, academic or family issues, there may be increasing blood glucose related concerns, especially hypoglycaemia and hyperglycaemia [24]. In fact, hypoglycaemia is described as a scary and frustrating moment which challenges optimal glycemic control. For this reason, many adolescents try to maintain higher blood glucose in order to avoid these situations [26, 30], despite concerns that premature death may be related to such poor glycemic control [24].

Once they enter into adult clinic setting, adolescent patients feel shocked by a bigger exposure to chronic diabetes complications as they see them in elderly people [33]. This intensifies their feeling of illness [34] and sense of responsibility for self-care. The perception of managing their disease changes [26] [35], and they realize the challenge of combining it with daily life [23, 36, 37].

Patients often have more difficulty doing similar daily life activities than their non-diabetic peers [24, 30]. Sometimes, problems related to diabetes can cause absences that hinder progress in education and relationships with peers [26]. Patients express misunderstanding by parents and teachers who believe their disease is used as an excuse for dropping out of school [24]. Besides, once patients start to work, they also have to navigate between family needs, diabetes care and work commitments, and they lack the flexibility to attend medical appointments [26, 38]. In addition, college education years can be found as a period characterised by increased independence but also a decrease in their clinical follow-up. This could be caused by the feeling that some topics patients found relevant, like sexuality or alcohol intake, were not addressed by healthcare providers [33]. Moreover, moving out of the parental home can lead to poor results in glycemic control [30]. Patients need to take responsibility for their lives, and they are not used to it, so they can be careless with their diet [39].

Daily management of their disease can be associated with a restriction on activities that mismatches with their expectations of living a “normal life”. This may precipitate emotional burdens [23]. Those who prioritise self-management over other emerging competing demands are found to experience a successful healthcare transition [35].

Young women with T1DM perceive motherhood as one of the most relevant life transitions they can live through. It requires close control of blood glucose levels, and patients feel worried about the health of their babies, especially if they could have diabetes too. Besides, once they have delivered, patients have to balance their own diabetic care and the needs of the newborn [26].

For parents of a child with T1DM, the diagnosis of the disease and the onset of new responsibilities are accompanied by a deep emotional distress and are described as a life-changing moment [31, 32]. The optimal care of their child needs a lot of time and dedication [31]. Once the child enters adulthood and acquires more responsibilities in their own care, the role of the parents turns to be more supportive and advisory, a further transition [31]. Communication with other parents of children with T1DM can also be very helpful and reduce anxiety levels [25].

### Experiences and acceptability of care transition

The transition of care from paediatric to adult services usually occurs at late adolescence between 15 and 19 years old [27, 34, 36, 40]. Loss of access to paediatric services occurs abruptly, which leaves the patients unprepared and sad for the loss of connection with their established healthcare providers [23, 41–43]. Therefore, patients prefer being treated by healthcare professionals with whom they feel comfortable and connected [42]. In fact, patients usually express their worries about switching to new health providers and the resulting therapeutic relationships [43].

Patients describe their experiences with the paediatric outpatient clinics as comfortable places with frequent examinations and a close follow-up, where healthcare providers also have a strong relationship with the family members [30, 36, 44]. Otherwise, adult services are found to be less personal, and appointments were less frequent [41, 44]. The difficulty of getting personalised health advice increases if the patient has no guarantee of seeing the same healthcare provider during clinical appointments [28]. In fact, patients expressed their preference in meeting the same healthcare providers at every consultation, which increases the feeling of trust [44]. Also, when the transition occurs in the same health center where patients received paediatric care, the experience is smoother [29].

In general, patients declare dissatisfaction with the organisation of the transition from paediatric to adult care when they found that the information received was limited. This points to a major problem [33, 44]. Also, dissatisfaction is expressed by patients due to lack of accompaniment from paediatric teams and lack of transfer coordination [33]. The quality of the interaction with the health service is crucial too. Lack of individualisation and disturbances during patient visits in adult care are also barriers to acceptability of the transition [37, 39].

Parents find their involvement is needed, but it is not easy to accommodate within adult services, leading to concerns about being excluded from care [27]. However, their degree of worry is more related to their child’s self-management and skills to handle their diabetes regimen alone and not the type of health service they receive [25, 27, 32, 34].

### Implementation considerations

One of the main challenges health services have to face during the transition is the loss of adherence to adult care visits [42]. This disruption of care could be attributed to young adults’ idiosyncrasy or the barriers generated by the health system [28, 38]. Coordination of care, addressing information needs and person-centered care are mentioned as facilitators for the transition.

Formal coordination of care is not common and patients express their desire for more support in organising the first adult appointment, which is commonly delayed [33, 38, 42, 43, 45]. A general healthcare provider could establish continuity of care between services, as mentioned before, which is considered a proper method for preparing adolescents competently [29, 46]. Some patients suggest that adult providers should participate in the last visit with their paediatric provider and they should be able to visit the adult clinic before the transition occurs [44]. Some workers in paediatric care settings where there is no general healthcare provider ignore current strategies in adult care. This affects one of the main goals of the successful transition which is the accomplishment of adolescents’ needs. This could be solved by establishing contact and collaboration between services and having routine feedback after transition [46].

Many times the information received by patients about adult transition is too limited [44, 47]. Information needs to revolve around daily self-management of T1DM (insulin dosage and injections or pumps, managing hypo-hyper glycemia, carbohydrate intake…) and executive functioning skills (blood work, maintaining prescriptions) [35]. Patients suggest that the ideal moment for receiving information would be before leaving paediatric care [47]. By contrast, adult care providers, who emphasise the importance of exploring adolescents’ knowledge too, think that the appropriate time should be after they transition to adult care [46].

Diabetes specialists are commonly seen by patients and parents as the most trustworthy source of clinical information. However, there are numerous patients who have limited access to them [42]. As such, there are multiple alternative sources of information, like web-based ones, that could meet their needs during the transition period [28]. Furthermore, patients are used to navigating through online search engines and social media for diabetes-related information when healthcare professionals are not available [26, 28]. The internet not only provides a useful source of information, but also a consistent means of support between connected type 1 diabetes peers, maintaining a high level of autonomy and anonymity and increasing their sense of control [26]. However, patients feel suspicious about the credibility of web-based information sources and find it challenging to manage the enormous amount of information [28].

To help ease the transition, some patients propose a telephone advice line, run by diabetes educators or nurses [45, 48], or online support (e-mail mentoring or written materials) [23, 48]. Besides, they would like to be attended by healthcare professionals with expertise in insulin bombs so they can more efficiently solve their support needs [45]. Besides, once young patients leave the paediatric clinics, the first adult appointment should not be delayed too long [45]. A gap of less than 3–6 months between the last paediatric visit and the first adult one is considered appropriate [29]. Some authors propose establishing a relationship between paediatric and adult services through a common healthcare professional who can provide continuity between patients and services [27, 34, 41, 46]. Providers suggest creating special clinics for youths at the age of transition or including adult clinic’s nurses to prepare for the transition [46].

Person-centered communication between the healthcare professionals and young patients makes them feel they are treated considerately and aids effective communication [37]. Patients appreciate behaviours including listening, helping to plan, or empathy [25]. Attending consultations with a diabetes nurse specialist increases satisfaction compared to those who only met the physician [44]. Hence, patients usually prefer healthcare providers who are more encouraging of mutual decisions and are collaborative and dialogue-oriented [35]. Healthcare providers recognise the importance of the care transition, although they complain about the lack of structured tools available in practice [29]. That is why providers also express frustration in finding adequate strategies which could suit adolescent’s needs [46].

In adult care, the balance between adolescents and young adults’ autonomy and the support for their parents can create tensions. Many adolescents still receive help from their parents, and some do not manage their disease at all [34] [23, 31, 41]. As part of the transition to adult services, encouragement of patients’ independence is valuable, for example, independent attendance at appointments [34, 35, 41] as patients tend to be more passive when parents are present [39]. But it also creates some tensions as parents do not have access to the content of consultations between the patient and health providers in order to maintain confidentiality [34]. Some services help to balance these tensions by gradually making the patient attend on their own [31, 32, 34].

Also, patients can find great support in peers, and these relationships are described as a highly valuable source of information too[28, 47]. The opportunity to discuss similar experiences reduces the sense of isolation and positively impacts their emotional well-being [23, 25, 26, 28, 47]. So, patients can find a great source of support from peer mentoring with diabetic adults who previously navigated the transition [23, 33]. This level of understanding and support is rarely found with healthcare professionals [28].

### Equity

Included studies did not analyse the impact of transition of care in relation to gender, economic status, ethnic origins or other social determinants of health. Only differences in care between paediatric and adult treatment are considered.

In some places, there are more resources in paediatric care compared to adult care, so patients find difficulties in receiving proper care as the frequency of appointments in adult care decreases [27]. That creates an imbalance in access to a multidimensional care [39, 42]. Adult consultations are shorter, less flexible, harder to get an appointment and have longer waiting periods [27, 36, 42]. Furthermore, difficulties in recovering their medical history, moving to different places or lack of personal connection with the health provider are found as barriers to receiving satisfactory medical attention [23, 26, 27].

## Discussion

This study highlights the wide range of barriers and difficulties that adolescents and young adults with T1DM face during the transition from paediatric to adult care. This transition is simultaneous to life-changing processes, such as entering the work market or college and leaving the parental home, which impacts their health status. When transitioning from paediatric to adult care, adolescents are also engaged in a critical phase of gradually increasing independence.

Our review shows that transition of care is usually sudden and without planning. Few adolescents receive preparation in the process which can affect adherence [49]. Therefore, not only may patients feel unprepared, but parents also express concern about being excluded from decision-making regarding their children’s health [27]. The acceptability of the transition process is suboptimal; adult care is considered less personal, comfortable and trustworthy than paediatric care [23, 41] [43]. Patients also perceive a lack of flexibility and difficulties in access [29, 46]. It has been observed that those patients who remain in paediatric care longer or transition within the same health center have better results and perceptions [11, 29].

Some implementation considerations can be taken from the results of this review for the development of interventions to increase the acceptability of transitional care. Patients and parents favoured a person-centered transition of care by signaling preferences for communication, coordination and continuity of care. A close follow-up accompanied with empathetic communication, active listening and personalised advice has been related to more satisfying transitions [30, 36, 41, 44, 44].

However, the difficulty of experiencing an optimal transition increases if patients have no guarantee to see the same health provider during clinical appointments [28]. In fact, patients expressed their preference for maintaining the same healthcare provider over time as continuity increases the feeling of trust [44]. These aspects highlight the importance of not only prioritising continuity but also ensuring appropriate coordination of care during transition [29, 46].

Some potential limitations can be found in this study. Due to the need for rapid GPC recommendations, the literature search ended in 2021 which means that some relevant studies published later have not been included. Additionally, we did not search for grey literature which may have resulted in a selection bias as the review did not include any books, policy documents or guidelines. Only studies in Spanish and English were included. Likewise, most of the articles included were from western or English-speaking countries. Works in other languages and contexts could have shown a more diverse overview of the phenomenon of interest. Nevertheless, our results are adequate and coherent for those contexts with transition of care between paediatric and adult services for T1DM. We believe data is saturated for the main findings related to experiences, acceptability and implementation consideration.

Future research should focus on identifying effective interventions to support the transition of care among emerging adults to optimise health outcomes. Studies will need to contemplate the implementation considerations. A systematic review [50] found only one study comparing a discharge program intervention to usual care in T1DM. A previous randomised control trial [51] did not demonstrate differences in 12-month outcomes but confirmed the difficulties of transition of care showing a late start and low frequency of visits in adult care. Digital approaches and group visits have been suggested as possible interventions to improve traditional care [52]. Some digital interventions have already proven acceptable and effective in improving knowledge and health outcomes in adolescents with T1DM [53]. Also, as our results show, peer support is very valued in this age group. According to the American Diabetes Association, diabetes self-management education with peer support should be provided during the transition to adulthood as it can provide emotional, instrumental, informational and appraisal resources [54]. Nevertheless, more research is needed in relation to the impact of social determinants of health not only in relation to the transition to adult care but in the overall diversity of experiences of living as an adolescent and young adulthood with T1DM. A few studies point out that children from low-income families have worse health outcomes and report lower quality of life [55, 56] and are less adherent to self-management interventions [57].

During the care transition, adolescents with T1DM undergo a critical phase fraught with a wide variety of challenges which may contribute to a suboptimal disease control. Increasing care independence by fostering autonomy, balanced with parent and peer support, may achieve more satisfying care after transitioning to adult services. The transition process between paediatric and adult services can be improved by increased information and strengthening continuity of care.

## Supplementary Information

Below is the link to the electronic supplementary material.

Supplementary Material 1 (PDF 44.3 KB)

## Data Availability

No datasets were generated or analysed during the current study.
