# Supplementary material for: Experiences and perspectives during the transition from paediatric to adult care in type 1 diabetes mellitus: systematic review of qualitative studies
Source: Eur J Pediatr. 2025 Jul 15;184(8):482. doi: 10.1007/s00431-025-06303-5 (PMC12263779; doi:10.1007/s00431-025-06303-5)
Supplement: Supplementary file 1 — Supplementary Material 1 (PDF 44.3 KB) [file 431_2025_6303_MOESM1_ESM.pdf]

Supplementary Material 1

Table S1. Medline search strategy

Database(s): Ovid MEDLINE(R) ALL 1946 to April 05, 2021		
#	**Searches**	**Results**
1	Diabetes Mellitus, Type 1/	77403
2	exp Diabetic Ketoacidosis/	6617
3	(diabet$ adj3 (britt$ or juvenil$ or pediatric or paediatric or child$ or early or keto$ or labil$ or acidos$ or autoimmun$ or auto immun$ or sudden onset or typ$ 1 or typ$ I)).ti,ab,hw.	115240
4	(insulin depend$ or insulindepend$ or insulin-depend$).ti,ab,kw.	29647
5	(IDDM or T1DM or T1D or dm1 or dm 1 or dmt1 or dm t1 or t1 dm).ti,ab,kw.	23501
6	1 or 2 or 3 or 4 or 5	134592
7	exp Diabetes Insipidus/	7968
8	diabet$ insipidus.tw.	8814
9	7 or 8	11075
10	6 not 9	134017
11	Adolescent/	2078645
12	(pediatric? or paediatric?).ti,hw.	235535
13	(adolescent? or adolescence or teen? or teenage or teenager? or juvenile or youth or young person? or young people or young adult? or young adulthood or young men or young women or young male? or young female?).ti,ab.	567567

14	Adolescent Medicine/	1535
15	Adolescent Health Services/	5689
16	Hospitals, Pediatric/	13877
17	11 or 12 or 13 or 14 or 15 or 16	2492247
18	transition*.ti.	95722
19	(transfer? or transferred or transferral or transferring).ti.	117295
20	(transition* adj10 (care or service? or center? or centre? or clinic? or facility or facilities or unit? or department? or patient?)).ab.	27337
21	((transfer? or transferred or transferral or transferring) adj10 (care or service? or center? or centre? or clinic? or facility or facilities or unit? or department? or patient?)).ab.	47485
22	(transition* and (adult? adj3 (care or service? or center? or centre? or clinic? or facility or facilities or unit? or department?))).ab.	2546
23	((transfer? or transferred or transferral or transferring) and (adult? adj3 (care or service? or center? or centre? or clinic? or facility or facilities or unit? or department?))).ab.	1072

24	(transfer? or transferred or transferral or transferring or transition).ab. and (adult?.ti. or (adult-focussed or adult-oriented).ti,ab.)	7246
25	(continuity adj3 (care or health care or healthcare or treatment? or therapy or therapies or patient? or doctor-patient or nurse patient)).ti,ab.	9974
26	"Continuity of Patient Care"/	19659
27	Patient Transfer/	8823
28	Patient Care Planning/	38922
29	"Delivery of Health Care, Integrated"/	13237
30	shared care.ti,ab.	1365
31	shared service*.ti,ab.	252
32	((healthcare or care or service*) adj3 integrat*).ti,ab.	25030
33	18 or 19 or 20 or 21 or 22 or 23 or 24 or 25 or 26 or 27 or 28 or 29 or 30 or 31 or 32	370343
34	17 and 33	29454
35	(adolescent medicine/ or adolescent health services/) and ((care or healthcare).hw. or (og or standards).fs.)	4024
36	(p?ediatric? adj2 adult?).ti,ab. and care.hw.	2298
37	34 or 35 or 36	34178
38	10 and 37	643
39	exp animals/ not humans/	4809293
40	"comment on".cm. or literature review.ti. or editorial.pt. or news.pt. or letter.pt.	2179425
41	38 not (39 or 40)	613

42	((("semi-structured" or semistructured or unstructured or informal or "in-depth" or indepth or "face-to-face" or structured or guide) adj3 (interview* or discussion* or questionnaire*)) or (focus group* or qualitative or ethnograph* or fieldwork or "field work" or "key informant")).ti,ab. or interviews as topic/ or focus groups/ or narration/ or qualitative research/	419376
43	41 and 42	92

Table S2. Web of Science search strategy

Database(s): WOS ALL 1900 to April 05, 2021		
**#**	**Searches**	**Results**
1	TS=(diabet* NEAR/3 ("type 1" OR "type i" OR britt* OR juvenil* OR pediatric OR paediatric OR early OR keto* OR labil* OR acidos* OR autoimmun* OR "auto immun*" OR "sudden onset"))	97.585
2	TS=((insulin* NEAR/2 depend*) or insulindepend*)	34.617
3	TS=(dm1 or "dm 1" or dmt1 or "dm t1" or t1dm or "t1 dm" or t1d or iddm)	29.809
4	#3 OR #2 OR #1	136.145
5	TS= "diabet* insipidus"	9.006
6	#4 NOT #5	135.908
7	TS= (pediatric? or paediatric?)	49.900
8	TS= (adolescent? or adolescence or teen? or teenage or teenager? or juvenile or youth or young person? or young people or young adult? or young adulthood or young men or young women or young male? or young female?)	1.046.353
9	#8 OR #7	1.088.456
10	TI= transition*	403.673
11	TI= (transfer? or transferred or transferral or transferring)	426.713
12	TS= (transition* NEAR/10 (care or service? or center? or centre? or clinic? or facility or facilities or unit? or department? or patient?))	33.564
13	TS= ((transfer? or transferred or transferral or transferring) NEAR/10 (care or service? or center? or centre? or clinic? or facility or facilities or unit? or department? or patient?))	44.971
14	TS= ((transfer? or transferred or transferral or transferring) and (adult? NEAR/3 (care or service? or center? or centre? or clinic? or facility or facilities or unit? or department?)))	266
15	AB=((transfer? or transferred or transferral or transferring or transition) and (adult ?.ti. or (adult-focussed or adult-oriented)))	183
16	TS= (continuity NEAR/3 (care OR "health care" OR healthcare or treatment* or therapy or therapies or patient* or "doctor-patient" or "nurse patient"))	9.776
17	ts= "shared care"	1.601
18	TS= "shared service*"	630
19	TS= ((healthcare or care or service*) NEAR/3 integrat*)	47.239
20	#19 OR #18 OR #17 OR #16 OR #15 OR #14 OR #13 OR #12 OR #11 OR #10	933.744
21	#20 AND #9 AND #6	405
22	TS=(qualitative OR ethnol* OR ethnog* OR ethnonurs* OR emic OR etic OR leininger OR noblit OR "field note*" OR "field record*" OR fieldnote* OR "fie ld stud*" or "participant observ*" OR "participant observation*" OR hermaneutic * OR phenomenolog* OR "lived experience*" OR heidegger* OR husserl* OR	1.671.533

	"merleau-pont*" OR colaizzi OR giorgi OR ricoeur OR spiegelberg OR "van kaam" OR "van manen" OR "grounded theory" OR "constant compar*" OR "theoretical sampl*" OR glaser AND strauss OR "content analy*" OR "thematic analy*" OR narrative* OR "unstructured categor*" OR "structured categor*" OR "unstructured interview*" OR "semi-structured interview*" OR "maximum variation*" OR snowball OR audio* OR tape* OR video* OR metasynthes* OR "meta-synthes*" OR metasummar* OR "meta- summar*" OR metastud* OR "meta-stud*" OR "meta-ethnograph*" OR metaethnog* OR "meta-narrative*" OR metanarrat* OR " meta-interpretation*" OR metainterpret* OR "qualitative meta-analy*" OR "qualitative metaanaly*" OR "qualitative etanaly*" OR "purposive sampl*" OR "action research" OR "focus group*" or photovoice or "photo voice" or "mixed method*")	
23	#22 AND #21	71

Table S3. EMBASE search strategy

Database(s): EMBASE ALL 1974 to April 05, 2021		
**#**	**Searches**	**Results**
1	'insulin dependent diabetes mellitus'/exp OR 'insulin dependent diabetes mellitus'	344,988
2	'diabetic ketoacidosis'/exp	12,509
3	(diabet* NEAR/3 (britt* OR juvenil* OR pediatric OR paediatric OR child* OR early OR keto* OR labil* OR acidos* OR autoimmun* OR 'auto immun*' OR 'sudden onset' OR 'type 1' OR 'type i')):ti,ab,de	128,326
4	((insulin* NEAR/2 depend*):ti,ab,de) OR insulindepend*:ti,ab,de	355,947
5	iddm:ti,ab,de OR t1dm:ti,ab,de OR t1d:ti,ab,de OR dm1:ti,ab,de OR 'dm 1':ti,ab,de OR dmt1:ti,ab,de OR 'dm t1':ti,ab,de OR 't1 dm':ti,ab,de	37,02
6	#1 OR #2 OR #3 OR #4 OR #5	408,209
7	'diabetes insipidus'/exp	14,89
8	'diabet* insipidus':ti,ab,de	15,752
9	#7 OR #8	16,298
10	#6 NOT #9	404,156
11	'child health care'/exp	96,195
12	'adolescent'/exp	1,649,986
13	pediatric?:ti,de OR paediatric?:ti,de	95,776
14	adolescent?:ti,ab OR adolescence:ti,ab OR teen?:ti,ab OR teenage:ti,ab OR teenager?:ti,ab OR juvenile:ti,ab OR youth:ti,ab OR 'young person?':ti,ab OR 'young people':ti,ab OR 'young adult?':ti,ab OR 'young adulthood':ti,ab OR 'young men':ti,ab OR 'young women':ti,ab OR 'young male?':ti,ab OR 'young female?':ti,ab	586,027
15	'pediatric hospital'/exp	22,85
16	#11 OR #12 OR #13 OR #14 OR #15	2,104,961
17	transition*:ti	87,349
18	transfer?:ti OR transferred:ti OR transferral:ti OR transferring:ti	9,216
19	(transition* NEAR/10 (care OR service? OR center? OR centre? OR clinic? OR facility OR facilities OR unit? OR department? OR patient?)):ab	33,049
20	((transfer? OR transferred OR transferral OR transferring) NEAR/10 (care OR service? OR center? OR centre? OR clinic? OR facility OR facilities OR unit? OR department? OR patient?)):ab	27,819
21	transition*:ab AND ((adult? NEAR/3 (care OR service? OR center? OR centre? OR clinic? OR facility OR facilities OR unit? OR department?)):ab)	570
22	(transfer?:ab OR transferred:ab OR transferral:ab OR transferring:ab) AND ((adult? NEAR/3	102

	(care OR service? OR center? OR centre? OR clinic? OR facility OR facilities OR unit? OR department?)):ab)	
23	(transfer?:ab OR transferred:ab OR transferral:ab OR transferring:ab OR transition:ab) AND (adult?:ti,ab OR 'adult focussed':ti,ab OR 'adult oriented':ti,ab)	11,845
24	(continuity NEAR/3 (care OR 'health care' OR healthcare OR treatment? OR therapy OR therapies OR patient? OR 'doctor patient' OR 'nurse patient')):ti,ab	11,784
25	'patient care'/de	292,733
26	'patient care planning'/exp	30,087
27	'integrated health care system'/exp	11,058
28	'shared care':ti,ab	2,05
29	'shared service*':ti,ab	329
30	((healthcare OR care OR service*) NEAR/3 integrat*):ti,ab	31,244
31	#17 OR #18 OR #19 OR #20 OR #21 OR #22 OR #23 OR #24 OR #25 OR #26 OR #27 OR #28 OR #29 OR #30	503,952
32	#16 AND #31	39,122
33	'child health care' AND (care:kw OR healthcare:kw OR og:kw OR standars:kw)	2,568
34	((p?ediatric* NEAR/2 adult*):ti,ab) AND care:de	1,404
35	#32 OR #33 OR #34	42,124
36	#10 AND #35	1,432
37	'animal'/exp NOT 'human'/exp	5,437,883
38	'comment on':it OR 'literature review':ti OR editorial:pt OR news:pt OR letter:pt	44,871
39	#37 OR #38	5,482,400
40	#36 NOT #39	1,507
41	(((('semi structured' OR semistructured OR unstructured OR informal OR 'in-depth' OR indepth OR 'face-to-face' OR structured OR guide) NEAR/3 (interview* OR discussion* OR questionnaire*)):ti,de) OR (focus:ti,de AND group*:ti,de) OR qualitative:ti,de OR ethnograph*:ti,de OR fieldwork:ti,de OR 'field work':ti,de OR 'key informant':ti,de OR 'qualitative research'/exp) AND ([english]/lim OR [spanish]/lim) AND ('article'/it OR 'letter'/it OR 'note'/it OR 'review'/it OR 'article in press'/it)	181,768
42	#40 AND #41	63
